# Being Evidence Based in Library and Information Practice

**DOI:** 10.5195/jmla.2018.348

**Published:** 2018-01-02

**Authors:** Claire B. Joseph

The modern evidence-based practice movement owes a lot to librarians; in fact, some would say that it could not have happened without them. Yet the library profession still has not totally embraced the concept of evidence-based practice in its own field, nor fully utilized it as a source for decision making. In this work, Koufogiannakis and Brettle bring together “recent theory, research and case studies from practice environments across the broad field of librarianship to illustrate how librarians can incorporate the principles of evidence-based library and information practice (EBLIP) into their work” (p. 3).

While the EBLIP movement was mentioned in 1995 by Haines and Roddham, Bradley, and Marshall, it was Eldredge who in 1997 not only first used the term “evidence-based librarianship,” but also firmly planted and started the movement in earnest. In 2000, the Medical Library Association Research Section created an Evidence-Based Librarianship Implementation Committee to promote EBLIP as both a practice and a part of the professional culture.

Evidence-based practice in libraries has evolved over the years and has become known as EBLIP. The model used in this book is based on Koufogiannakis’s doctoral research results. Koufogiannakis and Brettle refer to Eldredge’s original model for EBLIP from 2000, as well as Booth and Brice’s 2004 work. Koufogiannakis’s “revised model for EBLIP” is described as one that takes a “holistic approach,” “incorporating research evidence as well as local evidence and professional knowledge” (p. 13).

An essential part of EBP is research that builds a body of knowledge, but “within librarianship…we also need to consider local context and circumstances, because the decision being made is specific to those circumstances. The populations we serve and their needs are not necessarily the same in all instances” (p. 14).

This work is divided into two parts. In part 1, the authors devote chapters to each of the five elements of their new model for EBLIP. Table 2.1: “Elements of the EBLIP process” (p. 15) outlines the five elements:

1. Articulate: A clear understanding of the problem or question must be reached. Part of articulating the problem includes working out what is known already and why the information is needed, as well as ensuring that the problem is set in the appropriate context.
2. Assemble: Evidence should be assembled from multiple sources that are the most appropriate to the question or problem at hand and should include research evidence, local evidence, and professional knowledge.
3. Assess: Evidence should be assessed for its quality (often known as appraisal or critical appraisal), determining what the evidence says as a whole.
4. Agree: Determine a course of action and begin implementation of the decision. If working with a group, try to achieve consensus based on the evidence and organizational goals.
5. Adapt: Evaluate the decision and how it has worked in practice. Revisit goals and reflect on the success of implementation.

Part 2 of this work, “EBLIP in Action,” offers all-important “real life” examples of EBLIP in all types of libraries. Wilson discusses “Practitioner-Researchers & EBLIP” and describes, among other issues, the challenges, motivation, funding, and ultimate benefits of conducting research as a practicing librarian, and their deep connection to EBLIP as they bridge the gap between research and practice.

Somerville and Kloda speak to EBLIP in “Academic Libraries.” They briefly summarize three widely diverse examples of EBLIP in academic libraries from around the world that “support the customization of EBLIP principles and practices to local circumstances.”

Eldredge, Marshall, Brettle, Holmes, Haglund, and Wallace devote a chapter to EBLIP in “Health Libraries,” where the movement found its genesis: “The EBLIP movement has helped to move research and its application into the practice realm…Health librarians will, no doubt, continue to pursue EBP in a way that includes the priorities and approaches common to the health settings in which they work” (p. 131). Other chapters speak to EBLIP in public libraries, school libraries, and special libraries. A number of figures and tables effectively amplify the text.

Koufogiannakis and Brettle present an excellent overview of the history and evolution of library evidence-based practice along with the current state of EBLIP. The model they have presented is demonstrated to be usable across the library spectrum. This work is highly recommended.

